# The emerging roles of bacterial proteases in intestinal diseases

**DOI:** 10.1080/19490976.2023.2181922

**Published:** 2023-02-26

**Authors:** Alberto Caminero, Mabel Guzman, Josie Libertucci, Alan E. Lomax

**Affiliations:** a Department of Medicine, Farncombe Family Digestive Health Research Institute, McMaster University, Hamilton, Ontario, Canada; b Gastrointestinal Diseases Research Unit, Kingston General Hospital, Queen’s University, Kingston, Ontario, Canada

**Keywords:** Protease, microbiota, pain, mucosal barrier, gluten, gut-brain axis, gastroenteritis

## Abstract

Proteases are an evolutionarily conserved family of enzymes that degrade peptide bonds and have been implicated in several common gastrointestinal (GI) diseases. Although luminal proteolytic activity is important for maintenance of homeostasis and health, the current review describes recent advances in our understanding of how overactivity of luminal proteases contributes to the pathophysiology of celiac disease, irritable bowel syndrome, inflammatory bowel disease and GI infections. Luminal proteases, many of which are produced by the microbiota, can modulate the immunogenicity of dietary antigens, reduce mucosal barrier function and activate pro-inflammatory and pro-nociceptive host signaling. Increased proteolytic activity has been ascribed to both increases in protease production and decreases in inhibitors of luminal proteases. With the identification of strains of bacteria that are important sources of proteases and their inhibitors, the stage is set to develop drug or microbial therapies to restore protease balance and alleviate disease.

## Introduction

Proteases are a diverse group of enzymes that cleave peptide bonds.^1^ They are classified according to their catalytic mechanism into serine, cysteine, aspartic, glutamic, threonine and metalloproteases.^2^ These enzymes play multifunctional roles in essential physiological processes, including digestion of dietary proteins, apoptosis, cell differentiation, inflammation and nociception, to name a few.^3–6^ Proteases are tightly regulated to prevent excessive degradation of host proteins or inappropriate immune activation, and imbalances between proteolytic and anti-proteolytic activity have been described in patients with different gastrointestinal (GI) disorders. For example, colonic tissues from patients with inflammatory bowel disease (IBD) and irritable bowel syndrome (IBS) have increased serine proteolytic activity (PA),^7,8^ suggesting a role in the pathophysiology of the disease.

In the past, most investigations in IBD and IBS have focused mainly on studying proteases released by the host. However, the gut hosts a vast and diverse microbial ecosystem, the microbiota, that has important impacts on human homeostasis and disease. The intestinal microbiota is a rich source of proteases, as microbes release different proteases for metabolism, defense, and host invasion. Gut microbes also produce protease inhibitors and protease-degrading enzymes, reflecting the importance of tightly regulating proteolytic activity.^9^ In the gut, microbial proteases were first identified as virulence mechanisms of pathogens.^10,11^ Elevated PA was also observed in stool supernatants from IBD and IBS patients compared to samples from healthy subjects, suggesting that enhanced PA in IBD and IBS may be due to elevations in PA from both host and microbial origin.^12,13^

With the advent of omics-based technologies, including DNA sequencing^14^ and mass spectrometry,^15^ the identification of a large number of bacteria living in the gut and their metabolic products have opened new routes for studying their contributions to GI diseases.^16,17^ The recent insights into the interactions of the microbiota and their products with the host have strengthened the hypothesis that the proteolytic capacity of bacteria plays an important role in gut homeostasis and disease. For example, elastase-like activity of the pathogen *Pseudomonas aeruginosa* can lead to the production of peptides following gluten metabolism with increased immunogenicity in celiac disease (CeD) patients,^18^ and serine proteases from a consortium of gut bacteria modulate the excitability of nociceptors via activation of protease-activated receptor 4 (PAR-4).^19^ However, the mechanistic characterization of specific bacterial products such as proteases in gut disorders still represent an enormous challenge. With the accumulating evidence suggesting that bacterial proteases play a key role in GI diseases, in this review we aim to highlight some of the key findings about their involvement in the development of IBD, IBS,CeD and GI infections, and consider their physiological implications.

## Proteases: functions and classification based on their catalytic mechanism

Proteases are found in all forms of life and are vital for the survival of all organisms.

**Figure 1.**
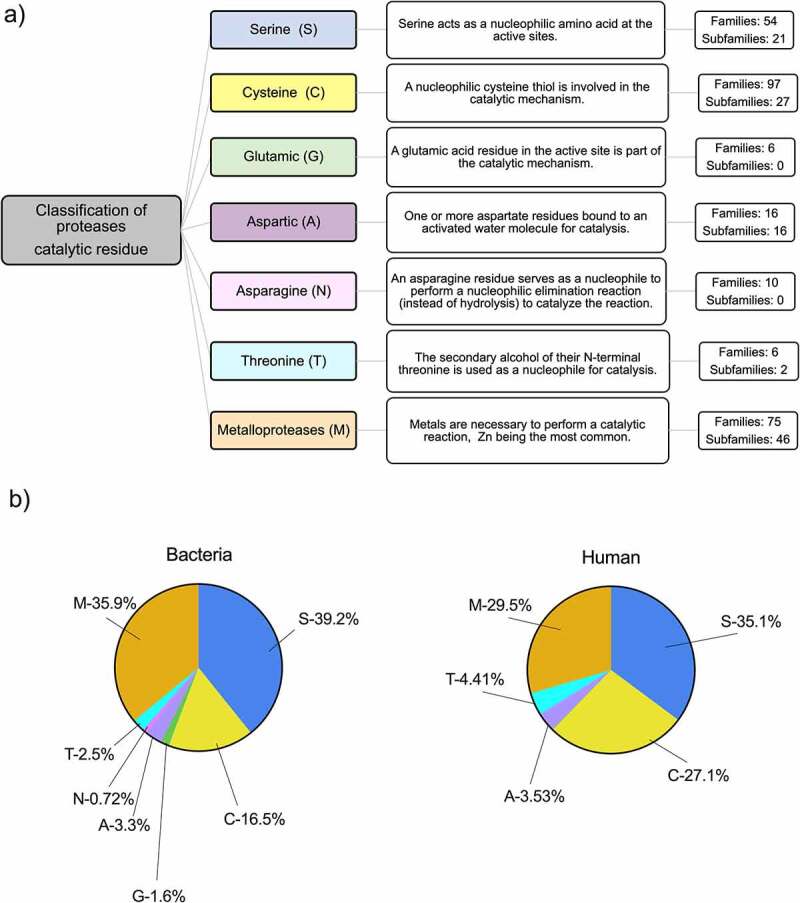
Classification and distribution of proteases based on their catalytic site. a) The MEROPs database classifies proteases based on the amino acid at the catalytic site used to carry out the catalytic process. Each group of proteases is identified by the letter of the amino acid that represents the catalytic type. All members are identified and classified based on structural similarities. b) Relative abundance of protease families in bacterial and human genomes, including putative proteases. Serine (S), cysteine (C), glutamic (G), aspartic (A), asparagine (N), threonine (T) and metalloproteases (M).

Based on their catalytic mechanism, they are classified into serine, threonine, cysteine, asparagine, glutamic, aspartic or metalloproteases (MEROPs database).^20^ These enzymes use an amino acid residue (serine, threonine, cysteine, asparagine, glutamic acid or aspartic acid, respectively) located in the active site to carry out the catalytic reaction, with the exception of metalloproteases, which use metals for catalysis, with Zn^2+^ being the most common (Figure 1a). In humans, proteases are one of the largest enzyme families, representing up to 2% of the human genome with over 500 different proteases described. Serine (35.1%) and metalloproteases (29.5%) are the most densely populated classes (Figure 1b).^21^ In addition to their well known functions in meal digestion, host proteases play very important roles in the gut, including cell proliferation and differentiation, tissue morphogenesis and remodeling, angiogenesis, wound repair, stem cell mobilization, inflammation, immunity, autophagy and apoptosis.^21,22^

Bacterial proteases are mechanistically, structurally and functionally highly diverse compared to mammalian proteases. Microorganisms produce a vast array of proteases belonging to the same classes described for humans in addition to glutamic-proteases, which have not been found in mammals so far. The most abundant bacterial proteolytic enzymes are serine (39.2%), metalloproteases (35.9%) and cysteine (16.5%) proteases (Figure 1b). Although belonging to similar classes, bacterial proteases have different functional activity or substrate specificity and not always are well-characterized. Indeed, domain architectures of many protease classes identified in bacteria are very different from those observed in eukaryotes, suggesting distinct roles for proteases in prokaryotes.^21,23^ In addition, the type and proportion of the different proteases vary between taxonomic groups and strains. From a functional point of view, proteases play important roles in intercellular communication, cell viability, stress response and pathogenicity in bacteria.

Bacterial proteases are better classified based on functional location as 1) cell-associated protease complexes and 2) extracellular enzymes.^24^ The first group includes intracellular, conserved and highly regulated proteases which are in multimeric complexes and essential for cell viability. These proteases are ubiquitous within the eubacteria kingdom and include the serine proteases Clp, Lon and high-temperature requirement serine protease A (HtrA), Zn^2+^ metalloproteinase FtsH and threonine protease HslUV. With the exception of the HtrA, these proteins fall into the broad class of AAA+ enzymes (ATPases associated with diverse cellular activities). ^24–26^ On the other hand, extracellular enzymes are generally monomeric with high substrate specificity. They are often synthesized as inactive zymogens, protecting the cell from unregulated activity before secretion. Many of these secreted proteases are considered virulence factors and are unique to certain taxon or strains.^11,24,25^ Many of them have been linked to different GI disorders as we will discuss in the next section.

## Involvement of bacterial proteases in gastrointestinal diseases

### Inflammatory bowel disease

IBD is an umbrella term that encompasses several diseases associated with chronic relapsing and remitting inflammation of the GI tract. The two major subtypes of IBD are Crohn’s disease (CD) and ulcerative colitis (UC). Although there are differences in the nature and location of gut inflammation between these two diseases, they share several debilitating symptoms that include pain, altered bowel habits, weight loss and anemia. Despite the availability of drugs and monoclonal antibodies that target key inflammatory processes to induce remission, many IBD patients are unresponsive or lose responsiveness over time. Therefore, novel therapies for IBD are needed, and one promising candidate approach is to target luminal proteolytic activity.

A dysregulation of host proteolytic has been well characterized previously in IBD patients.^8^ In both CD and UC patients, increased host serine protease production from colonic tissue have been reported.^27,28^ Proteases such as cathepsin G and thrombin are overactive in supernatants of colonic tissues from IBD patients compared to healthy controls.^8^ Elastase-like activity has also garnered considerable attention in IBD. Motta *et al*.^29^ showed that colonic epithelial cells are a major source of elastase-like activity and this activity is markedly increased in IBD patients. The same study specifically identified elastase 2A (ELA2A) as enhanced in IBD patients. Protease activity within fecal samples from patients has been also tested and revealed an increase in serine protease activity during IBD.^12^ Unlike colonic tissue, fecal samples contain proteases from both the host and bacteria, and deciphering the origin of the proteases found in fecal samples remains a challenge. Consistent with a role of proteolytic overactivity in disease pathogenesis, inhibitors of serine proteases have beneficial effects in mouse models of IBD.^27,30^ Elafin, a *Lactobacillus*-derived inhibitor of serine proteases reduced the severity of colitis in mice exposed to dextran sulfate sodium (DSS) or trinitro benzene sulphonic acid.^27,30,31^

In 2021, Galipeau and colleagues proposed the use of bacterial proteases as a marker of disease in UC patients.^32^ In a longitudinal cohort of UC patients, it was found that increased fecal proteolytic activity was seen in UC patients, even prior to disease onset. This suggests that increased fecal proteolytic activity could be used as an early biomarker of disease. More importantly, enhanced fecal proteolytic activity may be an early step in UC pathogenesis, due to proteolytic effects on mucosal barrier function and immunoregulation. A bacterial source of this fecal proteolytic activity was suggested due to detection of an increase in bacterial protease gene expression by shotgun metagenomics. Authors also found an increase in fecal elastase-like activity in UC patients. Elastase-like activity was positively correlated with the relative abundance of *Bacteroides vulgatus*, a taxon known to have high proteolytic activity. Importantly, transfer of fecal microbiota from UC patients prior to disease onset into mice increased fecal proteolytic activity in the colon and activation of host inflammatory responses. These results suggest a bacterial contribution to fecal proteolytic activity and provides proof of concept that the proteolytic activity of UC patient microbiota is sufficient to induce gut inflammation. Findings from Galipeau et al., 2021 were recently confirmed by Mills and colleagues^33^ using proteomics and metabolomics, which can detect whether the proteases are of eukaryotic or prokaryotic origin. A subset of the clinically active UC patients had an overabundance of proteases derived from the bacterium *Bacteroides vulgatus*. Some of the correlated proteases included serine and metalloproteases that largely function in the extracellular space and may exacerbate disease activity. Taken together, there is evidence to suggest that an increase in bacterial proteases is associated with UC pathogenesis but the extent to which these proteases drive disease severity and whether a similar phenomenon occurs in CD remain to be determined.

### Celiac disease

CeD is a chronic autoimmune inflammatory enteropathy that occurs in genetically susceptible individuals in response to the ingestion of gluten proteins. It has a worldwide prevalence of 1.4%.^34,35^ CeD primarily affects the small intestine, with the development of a mucosal immune response characterized by an increase of intraepithelial lymphocytes, villous atrophy and crypt hyperplasia.^36^ Currently, the only accepted treatment for CeD is a strict, life-long gluten-free diet.^37^ The role of microbes in CeD has gained considerable attention recently, based on reported alterations of the intestinal microbiome of CeD patients and associations between enteric infections with CeD onset in longitudinal studies.^38–40^

Along with changes in microbial composition, several studies have suggested a pivotal role of proteases in CeD pathogenesis. Contrary to a beneficial role of proteases in CeD, an increase in proteolytic activity towards gluten proteins has been observed in the duodenum and feces of CeD patients.^41,42^ Although the nature of these proteases is not well understood, recent reports have suggested a microbial origin. Duodenal biopsies from patients with active CeD have increased proteolytic activity against gluten that correlated with increased abundance of *Pseudomonas*, a well-known proteolytic taxon. Indeed, *Pseudomonas aeruginosa* producing the metalloprotease LasB induced food sensitivity in preclinical mouse models by different mechanisms, as described below.^18^ These studies suggest that proteases expressed by pathobionts impact gluten metabolism and immune activation in the small intestine of CeD patients.^43^ Consistent with a pathogenic role of proteolytic activity in CeD, a serine protease inhibitor produced by *Bifidobacterium longum* reduces gluten-induced immunopathology in a preclinical mouse model.^44^

For decades, it was suspected that missing digestive proteases may be the cause of CeD.^45,46^ This theory posited that defective digestion of gluten in CeD due to the lack of unknown host digestive proteases in susceptible individuals. Indeed, oral enzymatic therapy, a widely investigated therapeutic approach in CeD, focuses on the digestion of immunogenic gluten peptides in the human GI tract through peptidase supplementation.^37^ In this regard, microorganisms from the oral cavity have emerged as candidates that have the potential to produce enzymes that degrade luminal gluten. *In vitro* studies have shown that proteases released by *Rothia* strains (*R. mucilaginosa* and *R. aeria)* are potential sources of gluten-degrading enzymes that specifically target immunodominan gluten peptides.^47^ Subsequently, the enzyme produced by *R. aeria* was isolated and identified as belonging to the S8 subtilisin protease family with high capacity to effectively degrade gluten.^48^ Other researchers have shown that Prolyl endopeptidases from *Flavobacterium meningosepticum, Sphingomonas capsulate* and *Myxococcus xanthus* have promising applications in the treatment of CeD. The recombinant proteins from these microbes have the capacity to break down gluten peptides with different subsite specificities.^49^ These findings suggest the promising potential of bacterial proteases in the treatment of CeD and some formulations have already entered phase II clinical trials.^37,50^ Taken together, the evidence to date suggests that some proteases protect against CeD and other exacerbate the disease, depending on the substrate specificity of the protease in question and its ability to reduce or increase the immunogenicity of gluten catabolic products. The next decade is likely to witness a significant increase in studies of proteases of bacterial origin that aid in the metabolism of gluten, since it is only partially digested by human digestive enzymes, regardless of the disease.^51^

### Irritable bowel syndrome

IBS is a common digestive disorder associated with chronic abdominal pain and altered bowel habit.^52^ Because IBS presents without the frank inflammatory damage that accompanies IBD, it is commonly viewed as a disorder of gut-brain communication. In addition to alterations in central nervous system processing of signaling from the gut, several changes within the bowel of IBS patients have been implicated in altered gut-brain communication, including altered serotonin release by enterochromaffin cells, altered mast cell-neuronal communication, and microbial dysbiosis.^53–56^ Similar to IBD, studies in mice and humans have provided evidence that host- and bacterial-derived proteases may contribute to pathogenesis and symptom generation.^7,13,57,58^ For example, Trypsin-like activity and tryptase release are increased in colonic biopsies from IBS patients.^7,58^ In a recent study, metagenomic analysis of faecal samples from patients with post-infectious IBS revealed an altered gut microbiota composition and high gut proteolytic activity driven by specific host serine proteases compared with controls. The authors also showed that β-glucuronidases released by commensal microbes suppressed host PA, thereby protecting the intestinal epithelium and suggest that a decrease in microbial β-glucuronidase activity may contribute to IBS pathogenesis.^57^ In agreement with a role for enhanced protease activity in IBS, nafamostat, an inhibitor of serine proteases, reduced visceral hyperalgesia in a rodent model of post-inflammatory IBS.^31^

## Gastrointestinal infections

GI infections account for a large burden of acute and chronic disease, and proteases are essential to the ability of many microorganisms to infect the host. Bacterial pathogens rely on proteolysis for variety of purposes during the infection process. Intracellular and membrane proteases such as Clp, Lon or HtrA contribute to virulence through the timely degradation of virulence regulators and indirectly by providing tolerance to adverse conditions in the host. In contrast, pathogen-dependent extracellular proteases facilitate host invasion by degrading host extracellular matrix components or by interfering with host cell and immune signaling, as we discuss in the next section.^11^ A good example is *Helicobacter pylori*, a bacterium that infects approximately half of the world’s population and is the leading risk factor for peptic ulcer disease and gastric cancer.^59^ Although different virulence factors have been described in *H. pylori*,^60^ the zinc-protease PqqE and the serine-protease HtrA disrupt gastric mucosal integrity^61–63^ and thereby facilitate bacterial invasion.

Proteases are also pivotal virulence factors of infectious agents associated with gastroenteritis. Gastroenteritis is a diarrheal disease characterized by an increase in bowel movement frequency and stool water content, with or without fever, vomiting, and abdominal pain.^64^ Diarrhea caused by enteric infections is a major factor in morbidity and mortality worldwide. Although more than twenty microbial pathogens are known to cause acute gastroenteritis, several *Escherichia coli* strains are the most common, constituting a significant risk to human health and remaining an important cause of infant mortality in developing countries.^65,66^ This group of bacteria includes different pathotypes such as enterotoxigenic (ETEC), enteropathogenic (EPEC), enteroinvasive (EIEC), enterohemorrhagic (EHEC) or enteroaggregative *E. coli* (EAEC).^66^ Other clinically relevant microorganisms causing diarrhea are *Shigella* spp., *Salmonella* spp, *Campylobacter jejuni/coli* and *Vibrio cholera*.^65^ Enteric pathogens utilize a variety of sophisticated strategies to colonize the intestinal tract, evade the immune system, proliferate, and damage the host. Virulence factors related to these bacteria have a wide range of activities including adhesins, toxins, iron acquisition factors, lipopolysaccharides, polysaccharide capsules, invasins and proteases.

The serine protease autotransporters from enterobacteriacea (SPATE) constitute a superfamily of virulence factors. These are high molecular weight serine proteases generally secreted into the external milieu via the autotransport pathway and are highly prevalent among enteropathogens, including *Shigella, Salmonella, Citrobacter* and all *Escherichia coli* pathotypes. Several findings suggest that SPATEs degrade host intracellular or extracellular substrates, which trigger a variety of adverse effects on host cells.^67^ SPATEs can be classified in 2 types classes. Class-1 SPATEs target intracellular substrates, eliciting cytotoxic and endotoxin effects on the host.^67^ On the other hand, class-2 SPATEs seem to disrupt mucosal barriers and modulate the immune response by targeting host glycoproteins. In this class, the serine protease Pic produced by *E. coli* (EAEC), *Citrobacter* and *Shigella flexneri* is a virulence factor associated with adherence, colonization and evasion of the innate immune system.^68–71^ Class-2 SepA produced by *Shigella flexneri* and EAEC is also indispensable for barrier disruption. ^72^ Finally, the zinc metalloproteases StcE and SslE secreted by *E. coli* EHEC and EPEC/ETEC respectively, contribute to intimate adherence of these bacteria to host cells, a process that is essential for colonization.^73–78^

Other proteases have been described as virulence factors in gastroenteritis. As *H. pylori, Salmonella typhimurium* and *Campylobacter jejuni*, a bacterium responsible for foodborne infections, interact with the host cell epithelium and establish infection by HtrA.^79–81^ The extracellular Zn-dependent metalloprotease hemagglutinin (HA) also called vibriolysin, has been implicated in the pathogenicity of *Vibrio cholerae. V. cholerae* can cause cholera, a severe diarrheal disease that can be quickly fatal if untreated and is typically transmitted via contaminated water and person-to-person contact.^82^ Although the cholera toxin is the primary driver of the infection, vibriolysin presents a broad range of potentially pathogenic activities including degradation of the mucus barrier or disruption of epithelial tight junctions.^10,83^ Proteases could also mediate infections indirectly. This is the case of *Clostridioides difficile*, one of the leading causes of health-care-associated infections and diarrhoea in many countries. *C. difficile* causes mild to severe diarrhoea and can lead to life-threatening conditions such as colonic perforation, pseudomembranous colitis and toxic megacolon. The toxins A and B (TcdA and TcdB respectively) released by the pathogenic *C. difficile* are decisive for the infection. An internal Cys protease domain activates the toxin resulting in downstream effects on host cells.^84^ As proteases are essential to the ability of many bacteria to infect the host and cause disease, it has been proposed to block specific proteases to prevent common gastrointestinal infections; however, there are still no approved drugs with this mode of action.^25^

In the following sections, the pathophysiological consequences of proteolytic activity in GI diseases is discussed in the context of luminal actions, effects on mucosal barrier function, cell and immune signaling and impact on visceral sensation.

## Effects of bacterial proteases in the gastrointestinal tract

### Luminal actions of bacterial proteases: diet-microbiota interactions

Diet is a major driver of microbial composition and function.^85,86^ Our intestinal microbiota is capable of using different dietary components to generate microbial metabolites with bioactive properties.^87^ As happens in mammals, microbes use proteases to meet their nutritional amino acid requirements by hydrolyzing available proteins from the host or the diet. Consequently, microbial protease activity can be influenced by dietary choices. Patients with chronic inflammatory or functional GI conditions recognize diet as a driving factor in symptom onset/severity.^88–90^ Thus, diet should be considered when investigating the role of microbial proteases in inflammation or dysfunction. Indeed, there are multiple mechanisms by which microbial proteases could influence homeostasis through diet.

First, western diets are characterized by their high protein content, and many improperly digested dietary proteins are capable of inducing aberrant immune responses in the gut. ^51,91^ The functional diversity of the human gut microbiota implies a vast catalog of metabolic pathways that participate in the digestion of dietary components, even proteins that are difficult to digest by human enzymes.^92,93^ Thus, dietary proteins not used by the host become substrates for microbial proteases. This is particularly important in food sensitivities such as CeD.^94^ The main environmental trigger of CeD, gluten, is not fully digested by host digestive enzymes.^51^ It has been shown that the human gastrointestinal tract harbours bacteria with the capacity to metabolize gluten. These include commensal bacteria such as *Actinomyces, Bacillus, Rothia, Staphylococcus, Streptococcus, Lactobacillus* or *Clostridium* but also opportunistic pathogens such as *Pseudomonas aeruginosa*.^95–98^ Indeed, microbial proteases efficiently participate in gluten metabolism *in vivo* by modifying gluten’s mucosal absorption and immunogenicity, ultimately affecting the adaptive immune responses to gluten associated with CeD.^43^ In a recent study, microbial glutamate carboxypeptidase genes were associated with efficient gluten degradation.^38^ On the other hand, *Pseudomonas aeruginosa*, an opportunistic pathogen isolated from the duodenum of CeD patients, increased gluten immunogenicity by producing peptides that better translocate across the intestinal barrier and activate gluten-specific T-cells from CeD patients.^18^ *P. aeruginosa* degrades gluten through LasB, and this metalloprotease also leads to a gluten-independent upregulation of inflammatory pathways via protease-activated receptor (PAR)-2 activation. In mice expressing CeD risk genes, *P. aeruginosa* LasB synergizes with gluten to induce more severe inflammation that is associated with moderate villus blunting. Thus, the human intestine represents a rich source of microbial proteases helping in the digestion of common dietary proteins, which can increase or decrease their final immunogenicity.

A similar phenomenon has been demonstrated with other recalcitrant dietary proteins such as wheat amylase trypsin inhibitors (ATI). ATI are able to induce innate immune activation in the gut via toll-like receptor 4 activation, with downstream effects on intestinal inflammation and antigen sensitization.^91,99,100^ Gut microbial proteases are able to digest ATI, thereby reducing the intestinal dysfunction associated with wheat proteins.^101^ For example, *Lactobacillus* strains degrade both gluten and ATI peptides, reducing their immunogenic properties.^18,101^

In addition to the direct effects of microbial proteases on the host due to the catabolism of proteins, microbes release a plethora of metabolites impacting host homeostasis such as branched-chain fatty acids, amino acids, ammonia, phenols, hydrogen sulfide.^102,103^ Interestingly, tyrosine metabolites such as p-cresol and 4-ethylphenyl sulfate, which may contribute to gut-brain communication,are altered in IBS.^104–108^ Another bacterial metabolite, hydrogen sulfide, has multiple roles in physiological processes in the gut and has been linked to intestinal inflammation and colorectal cancer.^109–111^ Finally, tryptophan is a precursor for the synthesis of several important bioactive molecules, such as serotonin, melatonin, nicotinamide and vitamin B3, in addition to many other physiologically important intermediates.^112^ Tryptophan is an essential aromatic amino acid found in different dietary sources such as poultry, fish, oats, and dairy products. This unique amino acid can be metabolized by the gut microbiota into a range of indolic compounds, some of which can activate key homeostatic receptors such as the aryl hydrocarbon receptor (AhR) or the Pregnane X receptor (PXR).^87^ Indeed, these receptors have been implicated in intestinal inflammation and microbial tryptophan metabolism is altered in patients with IBD and CeD.^113–116^ Thus, microbial proteases could indirectly modulate different intestinal conditions by modifying common dietary antigens or facilitating the release of bioactive metabolites in the gut.

### Impact of bacterial proteases on mucosal barrier function

The first line of host defence against both commensal bacteria and invading enteric pathogens is the intestinal mucosal barrier, which is a physical barrier that includes both biochemical and immunological components. The physical barrier consists of epithelial cells connected by tight junctions and protected by an overlying host-secreted mucus layer.^117^ The mucus layer in the gut forms a physical barrier between host epithelial cells and the gut microbiota. There is a continuous turnover of the mucous layer and deficiencies in this dynamic system have been linked to gastrointestinal diseases and colonic cancer. The primary component of mucus is the gel-forming mucin 2 (MUC2) protein, which is synthesized by goblet cells. MUC2 deficient mice are more susceptible to developing spontaneous colitis^118^ and MUC2 gene levels were found to be altered in both UC and CD compared to healthy controls.^119,120^ Mucus organization in the colon is vastly different from that of the small intestine. The mucus in the small intestine forms a single and penetrable layer but the bacteria are kept away from the epithelium by antibacterial mediators. The mucus in the colon forms a double layer. The inner mucus layer is firmly attached to the epithelium, impenetrable to bacteria and essential for inhibiting the interaction of microbes with host receptors on the epithelium. The outer mucus layer in the colon (secreted) is expanded and serves as a habitat for the microbiota.^121^

Although a major function of secreted mucus is to protect the host epithelium from both commensals and pathogens, the glycoproteins in this barrier also create a nutrient source for some colonic microorganisms. Mucin provides carbon and nitrogen sources to bacteria and the exposed O-glycan chains serve as attachment sites for bacterial colonization. The commensal microbiota adhering to mucins protect the host through colonization resistance. For decades the idea that contents found in fecal samples can degrade colonic mucus has been discussed.^122,123^ The microbial carbohydrate-active enzymes (CAZymes) required for mucus degradation have been intensively studied in recent years, especially among members of *Bacteroides and Ruminococcus.^117^* Proteases from different microorganisms also exhibit strong proteolytic mucinase activity.^124^ Zinc metalloprotease ZmpB from *Clostridium perfringens* cleaves adjacent to glycosylated Serine and/or Threonine residues. Proteases from different infectious agents such as Pic in diarrheagenic *E. coli* and *Shigella*, or vibriolysin in *V. cholera*, degrade the colonic mucus, which is a key step facilitating epithelium invasion.*^10,^^68,^^69,^^71^* StcE and SslE, metalloproteases from diarrheagenic *E. coli* strains, also cleave mucin glycoproteins, which may help the pathogen to reach the epithelium.^74–76^ In addition, the M60-like protease family cleaves the mucin glycoprotein backbone in a manner that is dependent on the presence of specific glycan sidechain structures.^125,126^ Many pathogens express this family of proteases for host invasion. Different mucin-degrading proteases have been also described in *Bacteroides*, a common commensal of the human intestine. These include proteases in *B. thetaiotaomicron* (BT4244) or *Bacteroides caccae.^126^*,^127^ Although mucin degradative capacity is considered a virulence factor of many GI pathogens, the implications in specific chronic intestinal diseases are not yet well understood.

In addition to effects on the mucus layer, proteases have been shown to disrupt the epithelial component of the mucosal barrier. The epithelial barrier function requires a contiguous layer of cells as well as the tight and adheren junctions that seal the paracellular spaces between them. Compromised intestinal barrier function has been associated with a number of disease states, both intestinal and systemic.^128^ Oral administration of the bacterial serine protease SP-1, produced by *Clostridium spp* resulted in impaired epithelial barrier, altered microbiota community compostion, and exacerbated DSS-induced colitis.^129^ Host proteases such as chymase or ELA2A are able to cleave tight and adherens junction proteins including zonula occludens-1 or E-cadherin.^22,29,130^ Microbial proteases also cleave inter-enterocyte junctions. The metalloprotease GelE, produced by *Enterococcus faecalis*, degrades E-cadherin leading to loss of barrier function that is evident before inflammation in a mouse model of spontaneous colitis.^131^ Tight junctions are also targets of infectious agents such as *Pseudomonas aeruginosa* (through LasB),^132^ *H. pylori* (PqqE and HtrA),^61–63^ *C. jejuni* (HtrA), ^79,80^ *V. cholerae* (vibrolysin),^10,83^ *Shigella, Salmonella* or pathogenic *Escherichia coli* (SepA).^72,133^ Thus, microbes use proteases for host invasion with important implications in the gut. Alteration in tight and adherens junctions lead to increased paracellular permeability of the epithelial barrier which is a pathophysiological hallmark of IBD and IBS. As mucin- and junction-degrading bacteria can cause damage, these enzymes may provide targets for protease inhibitors to treat or prevent intestinal diseases.^25,117^

Another mechanism whereby luminal proteases can modulate the function of the epithelial barrier is via the activation of protease activated receptors (PARs) expressed on the membrane of enterocytes. PARs are a family of receptors with pleiotropic effects which have been implicated in different gastrointestinal conditions and is covered in detail in the sections below. Regarding the role of PARs in intestinal function, apical administration of PAR-2 activating ligands led to prostanoid and interferon release as well as an increase in paracellular permeability due to ZO-1 degradation.^6,134^ PAR-4 activation by cathepsin G, a protease that is elevated in fecal samples from UC patients, led to increased mucosal permeability and inflammation *in vivo* in mice.^28^ Mucosal damage and inflammation caused by toxin A from the enteric pathogen *C. difficile* is markedly reduced when PAR-2 activation is prevented.^135^ Although it is clear that luminal proteases have pronounced PAR-dependent effects on enterocyte function, additional work is required to determine the importance of the contribution of microbial proteases to these effects.

### Bacterial protease effects on cell and immune signaling

Proteases are signaling enzymes that can specifically regulate cell and immune signaling by different mechanistic pathways, including those mediated by PAR activation. PARs are G-protein coupled receptors that have seven transmembrane domains, an extracellular N-terminal and an intracellular C terminal. Proteolytic cleavage of the N-terminal initiates intracellular signaling by revealing a tethered ligand. The activation of the different members of the PAR family (PAR-1, 2,3 and 4) is protease specific, tightly regulated and influences a number of physiological functions in the gut such as motility, permeability and nociception.^22^ The functional consequence of a particular protease activating PARs depends on which PAR is activated and what its downstream signaling pathways are, including whether canonical or biased signaling pathways are initiated.^136,137^

PARs are ubiquitously expressed in the gastrointestinal tract (epithelial cells, neurons, mast cells, fibroblasts, etc) and mediate a wide array of pro-inflammatory, pronociceptive and proliferative effects after activation by proteases. PARs have been implicated in the pathogenesis of colorectal cancer^138^ and inflammatory and functional intestinal disorders.^139–141^ Moreover, elevated expression *of par2* in the colon tissue of patients with UC has been described. ^142^ Different microbial proteases have been proposed as activators PARs. Of relevance, LasB from *Pseudomonas aeruginosa*,^18,143^ and GelE from *Enterococcus faecalis*^144^ degrade the N-terminal of PAR-2, contributing to food sensitivities and intestinal inflammation in preclinical mouse models. However, the full implications of activating PARs by microbial proteases in the context of GI diseases are still being resolved.

Proteases are also able to stimulate or diminish the production of key host immune mediators such as cytokines or immunoglobulins (Ig). Cytokine production is a dynamic event that is tightly regulated. Disturbances in its dynamics can provoke exacerbated responses in the host as they are involved in multiple cascades of intracellular signaling.

For example, cleavage and activation of PAR-2 on human neutrophils by gingipain-R from *Porphyromonas gingivalis* induces the release of proinflammatory cytokines such as interleukin (IL)-6, IL-8 and tumour necrosis factor (TNF)-α.^145^ Interestingly, cytokines can also be degraded by bacterial proteases. Previous reports have shown that alkaline proteases and elastase from *Pseudomonas aeruginosa* can degrade IL-2 and interferon (INF)-γ.^146,147^ A Zn-metalloproteinase from *Legionella pneumophila* also has the ability to degrade IL-2.^148^ Likewise, gingipains, a trypsin-like cysteine proteinases produced by *Porphyromonas gingivalis*, can cleave IL-1β, IL-6, and IL-1ra.^149^ The same phenomenon can be observed with other protein-based mediators such as Ig. Ig are glycoproteins produced by plasma cells, which play an important role in adaptive immune responses by specifically recognizing particular antigens. In addition to the capacity of the microbiota to stimulate different Ig subtypes in the host, microbes can also degrade Ig helping immune system evasion. Many pathogens that infect mucosal surfaces encode proteases that cleave immunoglobulin such as *Neisseria meningitides, N. gonorrhoeae or Streptococcus pneumoniae*.^150,151^ It has been shown that the intestinal microbiota can degrade IgA, which plays a key role in mucosal immunology, and mice with documented degradation of secretory IgA are more susceptible to chemically-induced colitis.^152^ Host proteases such as trypsin are capable of degrading IgA, and microbial commensals such as *Paraprevotella* can prevent its degradation in the gut.^9^ Most studies have focused on understanding the interplay of bacterial exotoxins and the immune system to prevent disease. However, much remains to be done to understand whether proteases released by commensals promote or neutralize the production of anti- and pro- inflammatory mediators, respectively.

### Bacterial proteases and abdominal pain

Activation of PARs by host-derived proteases has been implicated in abdominal pain for the past two decades. Agonists of PARs excite spinal afferent neurons that innervate the GI tract.^153,154^ Biopsy supernatants from IBS patients have also been shown to excite spinal afferent neurons via PAR-2 activation.^7,58,155^ These excitatory effects have been ascribed to sensitization of transient receptor potential (TRP) channels, including TRPV1, TRPV4 and TRPA1 as well as suppression of voltage-gated K^+^ channels. There is also evidence from models of colitis that PAR-2 activation contributes to nociceptor hyperexcitability.^156^ Importantly, activation of PAR-4 has opposite effects on nociceptor activation to PAR-2 activation in rodents. PAR-4 activation suppresses the excitability of colonic nociceptors *in vitro*,^157^ and *in vivo*.^158^

A role for bacterial proteases in the modulation of abdominal pain was first suggested by *in vivo* experiments using fecal supernatants from patients with IBD or IBS by Bueno and colleagues. Abdominal pain sensitivity was measured in rats and mice by quantifying the visceromotor response to colorectal distension. Intracolonic administration of fecal supernatants from IBS-D and IBS-C patients increased the visceromotor response to distension, with evidence of both allodynia and hyperalgesia.^159,160^ In contrast, fecal supernatants from UC patients had the opposite effect, decreasing the visceromotor response in a PAR-4-dependent manner.^159^ Thus, it appears that fecal proteases can either exascerbate or suppress abdominal pain in rodents, depending on the relative amount of PAR-2 or PAR-4 activation that occurs.

Another important consideration is where the sites of action of fecal proteases are relevant to pain modulation. PARs are expressed on many cells within the wall of the gut, including spinal afferent neurons and enterocytes. Based on the study of fecal proteases from IBS-C patients, enhancement of abdominal pain did not appear to be due to direct excitatory actions of proteases on neuronal PARs.^160^ Instead, intracolonic administration of cysteine proteases within IBS-C fecal supernatants to mice increased colonic permeability and led to degradation of occluding, which in turn led to increased visceral pain. Mucosal biopsies from IBS-C patients also displayed evidence of epithelial occludin degradation compared to biopsies from healthy controls.^160^ However, because actions of fecal proteases on neuronal activation were not assessed in this study, it remains possible that direct effects on nociceptor nerve terminals in the gut neu also contributes to visceral pain, as the reduction of mucosal barrier integrity would facilitate access of luminal proteases to spinal afferent nerve terminals.

Subsequent studies have identified *Faecalibacterium prausnitzii* as a potential source of anti-nociceptive mediators, including a PAR-4 activating serine protease. Using two well-established rodent models of IBS that lead to visceral hyperalgesia *in vivo*, it was found that the enhanced visceromotor response to colorectal distension was reversed following administration of *F. prausnitzii*. .^161–164^These antinociceptive effects were due to a reversal of the increase in mucosal permeability that is a feature of these IBS models. *In vitro* experiments on dorsal root ganglion neurons also support an anti-nociceptive role of *F. prausnitzii.^19^* Media supernatant from cultures of *F. prausnitzii* acted directly on DRG neurons to suppress their excitability due to an increase in voltage-gated K^+^ conductance. This was the result of a cathepsin G-like serine protease that activated neuronal PAR-4.

In summary, PAR activation is able to suppress or augment abdominal pain, depending on which proteases predominate and which receptors they activate. Studies on samples from patients with abdominal pain indicate that both host and bacterial proteases could potentially contribute to pain. Given the evidence of luminal proteolytic imbalance in diseases associated with abdominal pain, including IBD and IBS, future studies directed towards further delineating the bacterial sources and cellular targets of these proteases will be valuable. The insights may lead to the development of next-generation probiotics that supress abdominal pain by shifting the balance of PAR activation to barrier-restoring and nociceptor-suppressing effects.

## Conclusions

**Figure 2.**
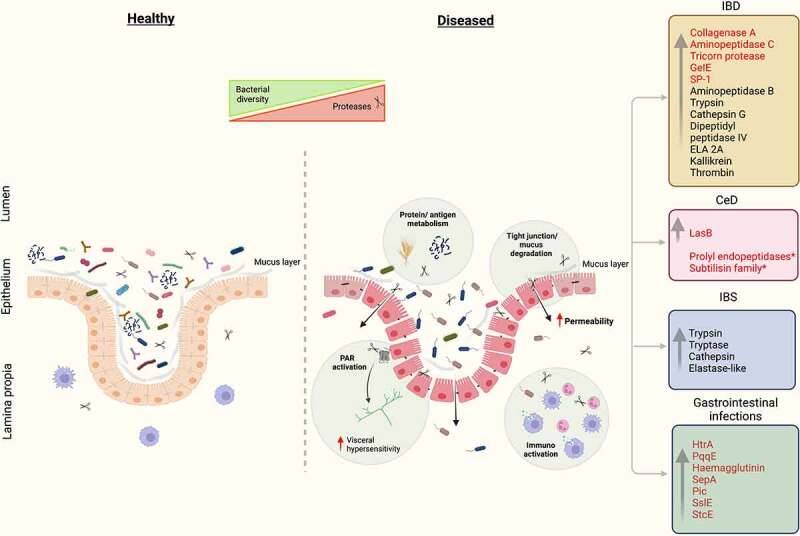
Proteases implicated in IBD, IBS, CeD and GI infections. In disease-related conditions, proteases induce structural and functional changes in the gut through multiple mechanisms of action, including effects on dietary protein metabolism, mucosal barrier function, neuronal excitability and immunoregulation. Luminal proteases impact GI function by a combination of PAR-dependent and independent effects. Proteases of microbial origin are highlighted in red. *Proteases with therapeutic potential. Figure was created with BioRender.com.

The impacts of proteases released by commensal bacteria on GI disease has received increasing attention in recent years. It has become evident that a complex balance between proteases, their host targets and protease inhibitors maintains the functionality and integrity of the gut. Dysregulation of this balance has a direct impact on intestinal health with serious consequences that lead to pathophysiological conditions (Figure 2). Furthermore, many pathogenic bacteria utilize proteases to colonise host tissues and cause disease (Table 1). Although recent findings have shown the importance of proteases from commensal gut bacteria in gut homeostasis, the study of these proteases and their contribution to disease is still in its infancy. However, as causal relationships between protease activity and disease are identified, along with mechanistic insights into how bacterial proteases promote or protect against disease, new opportunities to treat common GI diseases and infections may result.

**Table 1.** Summary of proteases of bacterial and host origin implicated in gastrointestinal diseases.

Protease	Source	Classification	Activity/mechanism	Disease	Ref
**Proteases released by bacteria**					
Collagenase A	*Clostridium perfringens*	Metalloprotease	Intestinal barrier alteration/mucus	IBD	^124^
Dipeptidyl peptidase IV Dipeptidase Amino peptidase C Tricorn protease homolog	Bacteroides/ *B. vulgatus*	Serine protease Metalloprotease Cysteine protease	Contribute to UC disease activity, barrier dysfunction	IBD	^32,33^
ND	*R. mucilaginosa*	ND	Gluten degradation	CeD	^47^
ND	*Lactobacillus*	ND	ATI peptides/gluten degradation	CeD	^101^
LasB	*P. aeruginosa*	Zn metalloprotease	Gluten degradation, PAR-2 mediated signaling Intestinal barrier alteration Degrade IL-2 and IFN-γ	CeD and others	^18,146,162^
Prolyl endopeptidases	*F. meningosepticum* *S. capsulate* *M. xanthus*	Serine peptidases	Gluten degradation	CeD	^49^
BAV86562.1 Subtilisin family	*R. aeria*	Serine protease	Gluten degradation	CeD	^48^
GeIE	*E. faecalis*	Metalloprotease	Compromise epithelial barrier	Colitis mouse model	^131^
SP-1	*C. ramosum*	Serine protease	Epithelial barrier dysfunction	Colitis mouse model	^129^
HtrA	*H. pylori* *C. jejuni* *S. Typhimurium*	Serine protease	Epithelial barrier alteration Growth and infection	Gastrointestinal infection	^63,80,81^
PqqE	*H. pylori*	Zn metalloprotease		Gastrointestinal infection	
Haemagglutinin or vibriolysin	*V. cholerae*	Metalloprotease	Barrier integrity alteration/actin and tight junction rearrangement	Gastrointestinal infection	^83^
SepA	*S. flexneri* *E. coli*	Serine protease	Barrier dysfunction/invasion	Gastrointestinal infection	^72^
Pic	*E. coli* *Citrobacter* *S. flexneri*	Serine protease	Disrupt mucosal barriers/modulate immune response/ mucinase activity	Gastrointestinal infection	^68–71^
SslE	*E. coli*	Zn metalloprotease	Mucin degradation	Gastrointestinal infection	^76^
StcE	*E. coli*	Metalloproteases	Mucus degradation	Gastrointestinal infection	^74,75^
ND	*L. pneumophila*	Zn Metalloproteases	Degrade IL-2	Gastrointestinal infections	^148^
PROKKA_00509	*Paraprevotella*	ND	Trypsin degradation	Others	^9^
M60-like proteases	*B. caccae*	Metalloproteases	Mucin degradation	Other	^127^
M60-like/PF13402 domain	*B. thetaiotaomicron*	Metalloprotease	Mucin degradation	Others	^125^
ZmpB	*C. perfringens*	Zn metalloproteases	Mucus degradation	Others	^117^
**Proteases released by the host**					
Aminopeptidase B	Human colonic tissues	Metalloprotease	Lysine-cleaving protease identified as active in CD	IBD	^8^
Cathepsin G	Human colonic tissue	Serine protease	Overactive	IBD	^8^
	Human stool		Epithelial barrier alteration	IBD	^28^
Dipeptidyl Peptidase-4	Human stool/intestinal tissue	Serine protease	Increased levels and expression	IBD	^163^
ELA 2A (Elastase 2A)	Human colonic cells	Serine protease	Epithelial barrier dysfunction	IBD	^29^
Thrombin	Human colonic tissue	Serine protease	Overactive	IBD	^8^
Trypsin-like, Elastase-like Cathepsin G-like Proteinase 3-like	Human stool	Serine proteases	Elevated activity	IBD	^12^
Kallikrein	Human intestinal tissue/stool	Serine protease	Inflammatory process in Crohn’s disease.	IBD/IBS	^13,164^
Trypsin-like Chymotrypsin-like Elastase-like	Human stool	Serine protease	Elevated in patients with high proteolytic activity	IBS	^13^
Trypsin-3/Trypsin-like activity	Human colonic tissue	Serine protease	Upregulated in intestinal epithelial, signal to enteric neurons and to induce visceral hypersensitivity/somatic and visceral hyperalgesia and allodynia in colon mice	IBS	^7,58^
Tryptase-alphabeta-1/Tryptase	Rat colonic tissue	Serine protease	Expression increased in colon of rats pots-colitis with visceral hypersensitivity/somatic and visceral hyperalgesia and allodynia in colon mice	IBS	_31_, _58_

ND = Not determined
